# Psychometric properties and comparison of four health utility approaches among myopia patients in China

**DOI:** 10.1186/s12955-023-02150-w

**Published:** 2023-07-04

**Authors:** Lei Dou, Yanjiao Xu, Gang Chen, Shunping Li

**Affiliations:** 1 Center for Health Management and Policy Research, School of Public Health, Cheeloo College of Medicine, Shandong University, Jinan, China; 2 NHC Key Lab of Health Economics and Policy Research, Shandong University, Jinan, China; 3 Center for Health Preference Research, Shandong University, Jinan, China; 4 Afliated Eye Hospital of Shandong University of Traditional Chinese Medicine, Jinan, China; 5 Center for Health Economics, Monash Business School, Monash University, Melbourne, Australia; 6 Center for Health Management and Policy Research, School of Public Health, Shandong University, Wenhua Xi Rd 44, Shandong Province 250012 Jinan, China

**Keywords:** Myopia, Health state utility, Health-related quality of life, Psychometric properties

## Abstract

**Background:**

The increased prevalence of myopia creates and earlier age of onset has created public health concerns for the long-term eye health, vision impairment and carries with it a significant economic burden. The quality of the economic evaluation is dependent on the sensitivity and validity of the approaches. Nowadays, there are many approaches to measure patients’ health state utility (HSU). However, little is known regarding the performance of direct approach and indirect approach in people with myopia. This study is aimed to compare the psychometric properties of four HSU approaches among patients with myopia in mainland China, including two direct approaches (TTO and SG), the generic preference-based measures (PBM) (AQoL-7D) and the disease-specific PBM (VFQ-UI).

**Methods:**

A convenience sampling framework was used to recruit patients with myopia who attended a large ophthalmic hospital in Jinan, China. Spearman’s rank correlations coefficient was used to assess concurrent validity. Known-group validity was analyzed by: (1) whether the patients wear corrective devices; (2) severity of myopia as low or moderate to high of the better eye; (3) duration of myopia as ≤ 10 years or > 10 years. Effect size (ES), relative efficiency (RE) statistic and the largest area under the receiver operating characteristic curve (AUC) were used to assess sensitivity. The intra-class correlation coefficient (ICC) and Bland–Altman plots were used to assess agreement.

**Results:**

A valid sample size of 477 myopia patients was analyzed (median duration: 10 years). The mean HSU scores between TTO and SG were similar (0.95) and higher than AQoL-7D (0.89) and VFQ-UI (0.83). Overall, the VFQ-UI had the best performance based on the psychometric analysis. The agreement indicated that there was no pair of approaches that could be used interchangeably.

**Conclusions:**

The VFQ-UI showed better psychometric properties than other three approaches for providing health state utility in Chinese myopia patients. Given the widespread use and its generic nature of the AQoL-7D, it could be used alongside with VFQ-UI to provide complementary health state utility from a generic and disease-specific perspective for economic evaluation. More evidence on the responsiveness of four health utility approaches in myopia patients is required.

**Supplementary Information:**

The online version contains supplementary material available at 10.1186/s12955-023-02150-w.

## Introduction

Myopia is the most common ocular abnormality in the world [1, 2]. It is estimated that the number of people around the world with myopia in 2016 is 1.406 billion (22.9% of the population), and it will increase to 4.758 billion people (49.8% of the world population) by 2050 [3]. The most rapid increases and highest prevalence have been recorded in East Asian countries, including China [4]. The estimated prevalence of myopia was about 80% in 18-year-old school children and 17% in adults aged 40 years and older in China [5]. Myopia, especially high myopia increases the risk of pathologic ocular changes such as cataract, glaucoma, retinal detachment, and myopic macular degeneration, all of which can cause irreversible vision loss [6]. The increased prevalence of myopia creates and earlier age of onset carries with significant economic burden [7, 8].

Cost-effectiveness analysis, especially cost-utility analysis (CUA), has been increasingly conducted to aid decision-makers concerning the allocation of scarce resources within healthcare [9]. The most widely used effectiveness measure in CUA is the quality-adjusted life years (QALYs) [10], and the key component of QALYs is the health states utility (HSU), which reflects the strength of an individual’s preference over the different quality of life dimensions [11]. The HSU, anchored on 0 (being dead) and 1 (full health), can be either measured directly approaches or indirectly approaches [9].

Standard gamble (SG) and time trade-off (TTO) are two popular direct approaches to measure HSU [11]. The indirect approach mainly refers to using preference-based measures (PBMs), which provide a standardized health state classification system and a tariff of quality weights for all health states described by the classification system [12]. Previous studies have shown that sometimes generic PBMs may not be sufficiently sensitive to vision-related quality of life [13, 14]. For example, the EQ-5D, lacks a vision-related domain and was insensitive to vision impairment and ocular disease [15–17]. Furthermore, evidence has shown that the PBMs that contain a vision dimension are more sensitive to measure the effects of visual impairment [18]. Therefore, the AQoL-7D which consisted six items for the vision dimension becomes the alternative choice [19]. In addition, given the generic PBMs have been shown to perform poorly in terms of sensitivity or responsiveness in vision impairment, disease-specific PBMs, the VFQ Utility Index (VFQ-UI) was developed to provide more sensitive preference-based estimates of health utilities for patients with varying levels of vision loss [20].

It has been proved that the different approaches of utility estimation yield different values, due to the difference in the dimensions described, the number of levels, and the severity range [21]. The quality of the economic evaluation is dependent on the sensitivity and validity of the approaches. Therefore, it is important to assess the validity of any health outcome instrument [13]. There have been some studies that compared validation between direct approaches (TTO and SG) [22], and among generic PBMs [13] in visual disorders. However, little is known regarding the performance of direct approach and indirect approach in people with myopia. The main aim of this study was to compare the psychometric properties of two direct approaches (TTO and SG), the generic PBM (AQoL-7D) and the disease-specific PBM (VFQ-UI) among myopia patients.

## Methods

### Study design and population

A cross-sectional survey was conducted with myopia patients from the Affiliated Eye Hospital of Shandong University of Traditional Chinese Medicine in China between September 2014 and March 2016. A convenience sampling framework was used to recruit myopia patients who were scheduled to undergo the LASIK (Laser- In Situ Keratomileusis) surgery. Patients whom spherical equivalent (SE) in both eyes of at least − 0.5 diopters (D) and did not have any other ocular disease were eligible to participate in this study. Patients were excluded if they were (a) unwilling to give informed consent, or (b) have a cognitive or intellectual impairment that could affect oral communication. This study was approved by the Ethics Review Board of the School of Public Health, Shandong University (Reference No.20141002), and the research adhered to the tenets of the Declaration of Helsinki.

After giving informed consent, a trained nurse administered the measures via face-to-face interviews in the waiting period between the routine eye examination and the refractive surgery-specific examination. During the interview, patients’ socio-demographic characteristics and HSU were obtained using a hardcopy questionnaire, while their clinical information was collected by the trained nurses from their visual tests and medical records.

### Measures

The questionnaire consisted of three sections. The first section included socio-demographic characteristics of patients. The second section included two direct approaches (TTO, SG), the generic PBMs (AQoL-7D), the disease-specific PBM (VFQ-UI) and the disease HRQoL instrument (NEI-VFQ-25). The third section included clinical characteristics of the respondents (e.g., spherical equivalent of better eye, duration of myopia and type of corrective devices).

#### TTO method

The TTO method measures the number of years the patient is willing to sacrifice for a new technology that restores prefect health [23, 24]. In this study, a direct TTO question previously used in China was adopted [25]. Participants were asked to predict their expected life expectancy and the maximum number of remaining years of life they would be willing to give up if they could receive an imaginary technology and have perfect vision in both eyes for the rest of their lives. The TTO utility score was calculated based on the time traded in years over the expected number of years of the respondent’s remaining life that (s)he is willing to give up for a hypothetical technology to restore perfect vision, that is:

$$\mathrm{TTO}\;\mathrm{utility}\:=\:1-\;\left(\mathrm{time}\;\mathrm{traded}\;\mathrm{in}\;\mathrm{years}\;/\;\mathrm{life}\;\mathrm{expectancy}\;\mathrm{minus}\;\mathrm{current}\;\mathrm{age}\right)$$

#### SG method

The SG is a method that has its theoretical basis in the von Neumann–Morgenstern axioms of expected utility theory. It aims at measuring the ‘disutility’ of a health state by observing the willingness to accept a certain risk of death in order to avoid the state [26]. In the study, participants were asked to consider a hypothetical scenario where a new treatment was developed that could give them perfect vision in both eyes for the rest of their life, but in this case there was an immediate risk of blindness if the treatment was unsuccessful. They were then asked what the maximum percentage risk of death, if any, they would be willing to accept. The SG utility score was calculated as the amount of risk (in percentage) of blindness that a participant is willing to take for the hypothetical technology that may restore perfect vision, that is:

$$\mathrm{SG}\;\mathrm{utility}\:=\:1-\;\left[\mathrm{amount}\;\mathrm{of}\;\mathrm{risk}\;\mathrm{of}\;\mathrm{blindness}\;\mathrm{in}\;\mathrm{percentage}\;\mathrm{that}\;\mathrm{the}\;\mathrm{participant}\;\mathrm{is}\;\mathrm{willing}\;\mathrm{to}\;\mathrm{take}/100\right]$$

#### AQoL-7D

The AQoL-7D is a comprehensive instrument created to increase the sensitivity of the measurement of quality of life amongst people with impaired vision. The descriptive system for the AQoL-7D was created by combining the descriptive systems of two extant generic instruments, the VisQoL and the AQoL-6D. It consists of 26 items which can be grouped into seven dimensions: independent living, relationships, mental health, coping, pain, senses and visual impairment [19]. The Chinese version of the AQoL-7D was adopted in this study and was scored using the original (and currently the only available) Australian tariff, with a theoretical utility ranged from 0 to 1 [19].

#### NEI VFQ-25

The National Eye Institute Visual Functioning Questionnaire-25 (NEI VFQ-25) is one of the most widely used of the visual function questionnaires [27]. It is a multidimensional questionnaire designed to assess visual disability and health related quality of life using 25 items across 12 subscales [28]. There are 12 subscales: 1 general health subscale and 11 visual functioning subscales, including general vision, ocular pain, color vision, near activities, distance activities, social function, mental health, role difficulties, dependency, driving and peripheral vision [29]. Items within each subscale are converted to a subscale score ranging from 0 to 100 and the overall composite score is calculated by averaging the 11 vision functioning subscale scores. A higher score indicates better vision-specific quality of life [30].

#### VFQ-UI

The Visual Function Questionnaire–Utility Index (VFQ-UI) is a vision-specific utility instrument that was calculated from the NEI VFQ-25 through application of the algorithm of Rentz et al. [31]. One item from each of six NEI VFQ-25 subscales (near activities, distance activities, social function, mental health, role difficulties, and dependency) was selected to develop a simplified eight vision-related health states classification (from best to worst function) using clinical input and Rasch analysis. Item response theory was used to derive the severity score (theta) for each state, and regression was used to map the severity score to a utility weight [20].

### Statistical analysis

#### Descriptive statistics

Descriptive statistics were performed on patients’ characteristics and the distribution of HRQoL scores. Categorical variables were calculated as frequencies and percentages, and continuous variables as means, standard deviation (SD) and median. Histograms were plotted for the four HSU values distribution. Continuous variables were tested for normality using the Shapiro–Wilk W test. All statistical analyses were performed using Stata software version 14.0 (StataCorp LP, College Station, Texas, USA) and MedCalc software version 16.8 (MedCalc Software, Ostend, Belgium). The level of significance was set at *p*-value ≤ 0.05 (two-tailed).

#### Concurrent validity

Concurrent validity assesses the strength of the relationship between measures of the same concept. In this study, concurrent validity was analyzed between four HSU measures and the disease-specific instrument NEI VFQ-25. Spearman’s rank correlations coefficient were calculated and correlation coefficients of ≤ 0.30 (weak), 0.30—0.49 (moderate), 0.50–0.69 (high), 0.70–0.89 (very high), ≥ 0.90 (nearly perfect) [32]. We formulated the following hypotheses based on the correspondence between the type of instruments and dimensions of respective instruments. First, there would be a weak correlation between the utility scores of the two direct approaches (TTO and SG) and two indirect instruments (AQoL-7D and VFQ-UI), as differ in questionnaires, respondents and hypothetical choice [33]. Second, compared with TTO and SG, we expected that AQoL-7D and VFQ-UI were moderate to strong correlate to NEI VFQ-25 composite score and subscales scores because both them included visual dimensions. Third, it was anticipated that VFQ-UI were more strongly correlate to NEI VFQ-25 than AQoL-7D, especially in six dimension (near activities, distance activities, social function, role difficulties, dependency, and mental health).

#### Known-group validity

Known-group validity assesses the extent to which scores on an instrument differ across groups in which they are expected to differ. In general, it is hypothesized that statistically significant difference in HSU scores would be detected between patients with different visual functioning status. Three groups’ comparisons were chosen based on previous research and clinical evidence. These were (1) whether the patients wear corrective devices (patients wear spectacles or contact lenses expected to have higher HSU scores than those without corrective devices) [25]. (2) severity of myopia as low ( SE ≤ -3.0D) or moderate to high ( SE > -3.0D) of the better eye (patients with poorer visual acuity expected to have worse HSU scores) [34]. (3) duration of myopia as ≤ 10 years or > 10 years (patients with longer duration expected to have worse HSU scores) [35]. Known-group validity was analyzed using the nonparametric Mann–Whitney U tests.

#### Sensitivity

The efficiency of the four health utility methods to detect clinically relevant differences of myopia patients were compared using effect size (ES), relative efficiency (RE) statistic and the largest area under the receiver operating characteristic curve (AUC) [36]. Cohen’s d was used to calculate standardized effect sizes. The Effect sizes was defined ≤ 0.5, 0.5 to 0.8 and ≥ 0.8 were small, moderate and large [32]. The RE was used to evaluate whether one instrument is more sensitive than another between groups of respondents known to differ and can be calculated using the ratio of F statistics between two instruments [36]. The coefficient higher than 1.0 indicates that the comparator measure is more sensitive than the reference measure at detecting clinically relevant differences and vice versa. The receiver operating characteristic (ROC) curve is a widely used method of evaluating the performance of measures against external indicators of health status. The AUC is regarded as the most sensitive as an instrument with ideal discriminative ability has an AUC of 1.0, and an AUC less than 0.5 means no discriminative power [37].

Since AUC analysis requires external criteria to be dichotomous, a focused literature search was performed to identify already established clinically meaningful severity cutoff points for the NEI-VFQ-25 instrument. Without an existing cutoff in the literature, we use the median scores to separate the patient sample into different severities in the main analysis. Across the head-to-head assessments, the frequency of having the strongest correlation coefficients, the largest AUC scores, and the largest absolute value of effect size were used to determine the best overall validity and sensitivity among four instruments. In addition, we also examined the ceiling and floor effect of the approaches, and it was considered to be present if more than 15% of respondents achieved the lowest or the highest possible score [38].

#### Agreement

Agreement between four HSU instruments was examined using intra-class correlation coefficient (ICC) and Bland–Altman plots. The ICC was computed with a two-way mixed effects model based on absolute agreement. Strength of agreement was based on the following thresholds: ICC = 0–0.2 (poor), ICC = 0.2–0.4 (fair), ICC = 0.4–0.6 (moderate), ICC = 0.6–0.8 (strong) and ICC > 0.8 (almost perfect) [39, 40]. The Bland–Altman plots illustrated the mean score between the two measurements [41]. The 95% limits of agreement (LOA) were bordered by ± 1.96 SD of the difference in the mean score between the two comparison instruments [42]. If there was good agreement between four approaches, then only 5% of points would lie outside of the LOA.

## Results

### Participants’ characteristics

Five hundred patients were invited to the interview. Among them, twenty-three participants who had missing values on key questions were excluded from this analysis, leaving a valid sample size of 477 patients (Table 1). The mean age was 25.2 years (range 16–48), males and females were equally divided. Of the participants, 35.4% were students and 94.3% completed high school and above education, and most of them (75.9%) lived in urban area. In terms of myopia, the refractive error of the myopia for both eyes ranged from -0.50 to -12.50D, and the mean refractive error of the myopia for better eye was -4.25 ± 1.85D (range -0.50 to -12.50D), and the mean duration of myopia was 10.6 ± 5.4 years. There were 81.4% participants wear corrective devices, including wear spectacles and contact lenses.

**Table 1:** Characteristics of participants (*N* = 477)

**Characteristic**	**N(%) or mean ± SD**
**Panel A–-Socio-demographic**	
**Age, years**	
Mean ± SD	25.2 ± 6.0
Range	16–48
**Gender**	
Male	240 (50.3)
Female	237 (49.7)
**Occupation**	
Students	169 (35.4)
Others	308 (64.6)
**Educational level**	
Primary or secondary school	27 (5.7)
High school or technical secondary school	107 (22.4)
Junior college	103 (21.6)
University degree and above	240 (50.3)
**Marital status**	
Married	167 (35.0)
Single	310 (65.0)
**Residence**	
Rural	115 (24.1)
Urban	362 (75.9)
**Panel B–- clinical**	
**Myopia in both eyes, D**	
Mean ± SD	-4.50 ± 1.88
Median	-4.25
Range	-0.50 to -12.50
**Myopia in better eye, D**	
Mean ± SD	-4.25 ± 1.85
Median	-4.00
Range	-0.50 to -12.50
**Duration of myopia, years**	
Mean ± SD	10.6 ± 5.4
Median	10
Range	1–30
**Type of corrective devices**	
Wearing spectacles	380 (79.7)
Contact lense	31 (6.5)
Both	25 (5.2)
Not wearing devices	41 (8.6)

Better eye, one eye with a lower degree of myopia for both eyes of participants

*SD* Standard deviation

### Descriptive statistic of approaches

**Fig. 1:**
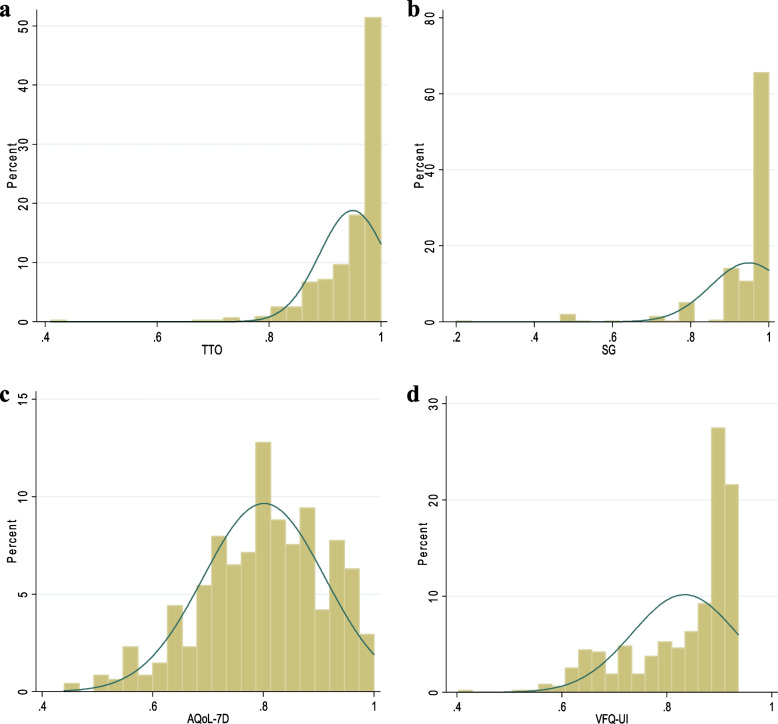
Distribution of health state utility scores elicited from four utility approaches. **a** Distribution of TTO health state utility score. **b** Distribution of SG health state utility score. **c** Distribution of AQoL-7D health state utility score. **d** Distribution of VFQ-UI health state utility score

The summary statistics of HSU scores and quality of life scores were presented in Table 2. The mean HSU scores between TTO and SG were similar (0.95) and higher than AQoL-7D (0.89) and VFQ-UI (0.83). The mean NEI-VFQ-25 composite score was 82.96. The distributions of HSU scores were shown in Fig. 1 which plots the histograms for each approach. The HSU scores of TTO, SG and VFQ-UI were all left-skewed, while the AQoL-7D was relatively close to a normal distribution. The participants’ HRQoL scores by each characteristic were shown in Supplementary Table 1.

**Table 2:** Descriptive statistics for five approaches scores in myopia patients

**Approaches**	**Theoretical range**	**Observed range**	**Mean (SD)**	**Median**	**Ceiling effect, N (%)**	**Floor effect, N (%)**
TTO	(0, 1.00)	(0.41, 1.00)	0.95(0.06)	0.98	36(7.5)	0(0)
SG	(0, 1.00)	(0.20, 1.00)	0.95(0.10)	0.99	98(20.5)	0(0)
AQoL-7D	(0, 1.00)	(0.44, 1.00)	0.80(0.11)	0.81	1(0.2)	0(0)
VFQ-UI	(0, 1.00)	(0.40, 0.94)	0.83(0.10)	0.88	0(0)	0(0)
NEI-VFQ-25	(0, 100.00)	(20.23, 100.00)	82.96(11.62)	85.11	3(0.6)	0(0)

Ceiling effect, % of respondents scored the highest possible health state; floor effect, % of respondents scored the lowest possible health state

*SD* Standard deviation

### Concurrent validity

Table 3 showed the correlations between four health utility approaches and NEI VFQ-25. All the hypotheses were accepted. The weak to high correlations were observed between the NEI VFQ-25 composite score and four HSU scores, ranging from 0.029 (SG) to 0.785 (VFQ-UI). As expected, the weak correlations between two direct approaches (TTO and SG) and NEI VFQ-25, while moderate to high correlations between two indirect approaches (AQoL-7D and VFQ-UI) and NEI VFQ-25. The strengths of correlations were stronger with VFQ-UI than AQoL-7D in general, and the strongest correlations were in the six dimensions (ranged from 0.556 to 0.716) of VFQ-UI. In summary, among four health utility approaches, the VFQ-UI exhibited the strongest correlations against the NEI-VFQ-25.

**Table 3:** Correlation between NEI VFQ-25 and four health utility approaches

**NEI-VFQ-25**	**TTO**	**SG**	**AQoL-7D**	**VFQ-UI**
Composite score	0.124^**^	0.029	0.493^**^	0.785^**^
General Health	0.073	0.029	0.328^**^	0.167^**^
General Vision	0.049	0.023	0.326^**^	0.338^**^
Ocular Pain	0.028	0.006	0.249^**^	0.266^**^
Near Activities	0.119^**^	-0.027	0.343^**^	0.716^**^
Distance Activities	0.136^**^	0.052	0.427^**^	0.641^**^
Social Function	0.151^**^	0.032	0.367^**^	0.665^**^
Mental Health	0.153^**^	0.099^*^	0.447^**^	0.642^**^
Role Difficulties	0.036	-0.008	0.283^**^	0.556^**^
Dependency	0.066	0.088	0.417^**^	0.647^**^
Driving	0.082	0.037	0.337^**^	0.468^**^
Color Vision	0.063	-0.017	0.203^**^	0.498^**^
Peripheral Vision	0.119^**^	0.043	0.322^**^	0.564^**^

*SD* Standard deviation; ** *p* < 0.01; * *p* < 0.05

### Known-group validity

Table 4 reported the known-group validity from the univariate analyses. Significant differences among known groups were found on severity of myopia and duration of myopia for VFQ-UI. As expected, patients with low myopia had significantly higher HSU (*P* = 0.001), and patients with shorter duration of myopia had significantly higher HSU (*P* = 0.000). There were no significant differences in whether wear corrective devices in all approaches.

**Table 4:** Known-group validity of four health utility approaches

**Groups**	**N (%)**	**TTO**		**SG**		**AQoL-7D**		**VFQ-UI**	
		**Mean (SD)**	***P*-value**	**Mean (SD)**	***P*-value**	**Mean (SD)**	***P*-value**	**Mean (SD)**	***P*-value**
**Severity of myopia**									
Low	138 (28.9)	0.95 (0.05)	0.761	0.94 (0.12)	0.278	0.81 (0.11)	0.200	0.85 (0.09)	**0.001**
Moderate to High	339 (71.1)	0.95 (0.06)		0.95 (0.09)		0.80 (0.11)		0.83 (0.10)	
**Duration of myopia (Years)**									
≤ 10	301 (63.1)	0.95 (0.05)	0.053	0.95 (0.10)	0.423	0.81 (0.10)	0.269	0.83 (0.10)	**0.000**
> 10	176 (36.9)	0.94 (0.07)		0.95 (0.10)		0.79 (0.12)		0.79 (0.12)	
**Wear corrective devices**									
Yes	436 (91.4)	0.95 (0.06)	0.796	0.95 (0.10)	0.169	0.80 (0.11)	0.810	0.84 (0.10)	0.617
No	41 (8.6)	0.95 (0.06)		0.94 (0.09)		0.80 (0.12)		0.82 (0.12)	

*SD* Standard deviation

### Sensitivity

Table 5 presents the ESs, RE statistics, and AUC scores for the four utility approaches. All the ESs of TTO and SG were less than 0.5, indicating the small discrimination ability. Most ESs of AQoL-7D were considered to be moderate except in the OP, RD, driving and CV subscales, while most ESs of VFQ-UI were considered to be large except in the GH, GV, OP and driving subscales. The calculated RE statistic showed that VFQ-UI had better sensitive than AQoL-7D, whereas TTO and SG is less sensitive than AQoL-7D. Furthermore, the AUC scores of AQoL-7D and VFQ-UI above 0.5 with statistical significance, which suggested that they are able to detect the difference between patients with severity of myopia. In the assessment of ceiling and floor effects (Table 2), the SG method showed a higher ceiling effect with 20.5%, and the TTO and AQoL-7D yield a small ceiling effect (7.5% and 0.2%), no floor effects were observed in all approaches.

**Table 5:** Efficiency of the four utility approaches to detect clinically relevant differences

**Groups**	**TTO**		**SG**		**AQoL-7D**		**VFQ-UI**	
	**Mean (SD)**	**ES**	**Mean (SD)**	**ES**	**Mean (SD)**	**ES**	**Mean (SD)**	**ES**
**NEI-VFQ-25 Total**								
< 85	0.95 (0.06)	0.042	0.95 (0.09)	0.064	0.76 (0.11)	0.850	0.77 (0.10)	1.806
≥ 85	0.95 (0.06)		0.95 (0.11)		0.84 (0.10)		0.90 (0.03)	
RE	0.002		0.006		1		4.496	
AUC	0.531		0.509		0.734*		0.924*	
**NEI-VFQ-25 GH**								
< 75	0.94 (0.06)	0.217	0.94 (0.10)	0.084	0.75 (0.11)	0.706	0.81 (0.11)	0.376
≥ 75	0.95 (0.06)		0.95 (0.10)		0.82 (0.10)		0.84 (0.09)	
RE	0.088		0.014		1		0.254	
AUC	0.564*		0.531		0.689*		0.605*	
**NEI-VFQ-25 GV**								
< 60	0.94 (0.09)	0.238	0.95 (0.09)	0.021	0.74 (0.11)	0.696	0.77 (0.10)	0.784
≥ 60	0.95 (0.05)		0.95 (0.11)		0.81 (0.11)		0.85 (0.09)	
RE	0.062		0.001		1		1.267	
AUC	0.534		0.517		0.701*		0.735*	
**NEI-VFQ-25 OP**								
< 87	0.95 (0.06)	0.025	0.95 (0.10)	0.039	0.77 (0.11)	0.473	0.80 (0.11)	0.587
≥ 87	0.95 (0.06)		0.95 (0.10)		0.82 (0.10)		0.85 (0.09)	
RE	0.003		0.007		1		1.281	
AUC	0.493		0.495		0.632*		0.654*	
**NEI-VFQ-25 NA**								
< 91	0.94 (0.07)	0.213	0.96 (0.08)	0.117	0.75 (0.11)	0.649	0.73 (0.10)	2.022
≥ 91	0.95 (0.05)		0.95 (0.11)		0.82 (0.11)		0.88 (0.05)	
RE	0.085		0.032		1		6.379	
AUC	0.565*		0.496		0.681*		0.894*	
**NEI-VFQ-25 DA**								
< 83	0.95 (0.06)	0.065	0.95 (0.09)	0.046	0.76 (0.10)	0.800	0.77 (0.11)	1.478
≥ 83	0.95 (0.06)		0.95 (0.11)		0.84 (0.10)		0.89 (0.05)	
RE	0.007		0.003		1		3.191	
AUC	0.529		0.511		0.722*		0.847*	
**NEI-VFQ-25 SF**								
< 100	0.94 (0.06)	0.160	0.95 (0.09)	0.007	0.76 (0.10)	0.747	0.76 (0.11)	1.580
≥ 100	0.95 (0.06)		0.95 (0.10)		0.83 (0.10)		0.89 (0.05)	
RE	0.046		0.000		1		3.753	
AUC	0.576*		0.517		0.710*		0.859*	
**NEI-VFQ-25 MH**								
< 81	0.94 (0.06)	0.174	0.95 (0.08)	0.006	0.75 (0.10)	0.928	0.76 (0.11)	1.495
≥ 81	0.95 (0.06)		0.95 (0.11)		0.84 (0.10)		0.88 (0.06)	
RE	0.035		0.000		1		2.054	
AUC	0.581*		0.557*		0.748*		0.851*	
**NEI-VFQ-25 RD**								
< 87	0.95 (0.06)	0.006	0.96 (0.08)	0.172	0.77 (0.11)	0.484	0.78 (0.11)	1.196
≥ 87	0.95 (0.06)		0.94 (0.11)		0.83 (0.10)		0.88 (0.05)	
RE	0.000		0.130		1		5.854	
AUC	0.501		0.475		0.642*		0.794*	
**NEI-VFQ-25 Dependency**								
< 91	0.95 (0.06)	0.098	0.95 (0.10)	0.047	0.75 (0.11)	0.782	0.77 (0.11)	1.429
≥ 91	0.95 (0.06)		0.95 (0.10)		0.83 (0.10)		0.88 (0.05)	
RE	0.016		0.004		1		2.662	
AUC	0.541		0.551		0.716*		0.825*	
**NEI-VFQ-25 Driving**								
< 75	0.95 (0.06)	0.103	0.95 (0.10)	0.009	0.78 (0.12)	0.391	0.80 (0.11)	0.694
≥ 75	0.95 (0.06)		0.95 (0.09)		0.82 (0.10)		0.87 (0.07)	
RE	0.070		0.001		1		3.077	
AUC	0.543		0.507		0.608*		0.692*	
**NEI-VFQ-25 CV**								
< 100	0.94 (0.06)	0.129	0.96 (0.09)	0.094	0.76 (0.11)	0.464	0.73 (0.11)	1.577
≥ 100	0.95 (0.06)		0.95 (0.10)		0.81 (0.11)		0.86 (0.07)	
RE	0.077		0.041		1		7.561	
AUC	0.540		0.486		0.635*		0.839*	
**NEI-VFQ-25 PV**								
< 100	0.94 (0.06)	0.167	0.95 (0.09)	0.028	0.77 (0.11)	0.627	0.77 (0.11)	1.356
≥ 100	0.95 (0.05)		0.95 (0.10)		0.83 (0.10)		0.89 (0.05)	
RE	0.071		0.002		1		4.252	
AUC	0.562*		0.519		0.674*		0.812*	

For the RE analysis, reference is AQoL-7D

*RE* Relative efficiency, *AUC* Area under the ROC curve, *ES* Effect size

*GH* General health, *GV* General vision, *OP* Ocular pain, *NA* Near activities, *DA* Distance activities, *SF* Social function, *MH* Mental health, *RD* Role difficulties, *CV* Color vision, *PV* Peripheral vision

^*^ *P* < 0.001

### Agreement

**Fig. 2:**
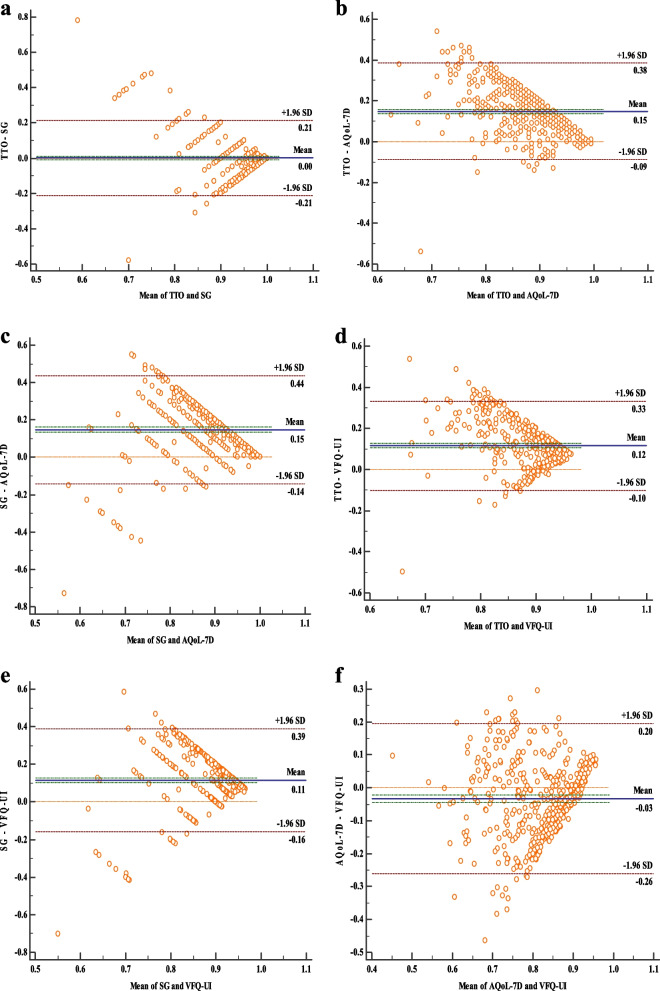
Bland–Altman plots between four health utility approaches. **a** Agreement between health state utility scores derived from TTO and SG. **b** Agreement between health state utility scores derived from TTO and AQoL-7D. **c** Agreement between health state utility scores derived from SG and AQoL-7D. **d** Agreement between health state utility scores derived from TTO and VEQ-UI. **e** Agreement between health state utility scores derived from SG and VEQ-UI. **f** Agreement between health state utility scores derived from AQoL-7D and VEQ-UI. The 95% limits of agreement are shown with a dashed line, and the mean difference between both measurements is shown with a solid line

The ICC value for pairwise comparisons ranged from -0.008 (AQoL-7D & SG) to 0.557 (AQoL-7D & VFQ-UI) (Table 6), indicating poor absolute agreements between each pair of utility approaches. The Bland–Altman plots of each pair of the approaches were presented in Fig. 2. As shown, the 95% limits of agreement among six pairs of comparison ranged from 0.42 to 0.58, which further indicated the poor agreements. Nevertheless, agreement between two direct approaches (TTO &SG) and two indirect approaches (AQoL-7D & VFQ-UI) were good.

**Table 6:** Agreements among four health utility approaches in myopia patients

**Approaches**	**TTO**	**SG**	**AQoL-7D**	**VFQ-UI**
TTO	1	0.111**	0.140	0.161
SG		1	-0.008	-0.003
AQoL-7D			1	0.557
VFQ-UI				1

^**^p < 0.01

## Discussion

To the best of our knowledge, this is the first empirical study to comprehensively assess and compare the psychometric properties of two direct approaches (TTO, SG) and two indirect approaches (AQoL-7D, VFQ-UI) among myopia patients. In most scenarios, the explored psychometric properties of the four health utility approaches were supported. As expected, the moderate to high correlations have been observed between disease-specific measures and the AQoL-7D and the VFQ-UI, while the weak correlations were observed with TTO and SG. Exceptions also exist, including (1) all the measures failed to distinguish differences between whether wear corrective devices in known-group validity (2) AQoL-7D cannot adequately reflect severity and duration of myopia. Based on the psychometric properties examined in this study, the indirect approaches outperformed direct approaches. Furthermore, the VFQ-UI demonstrated the best performance among four health utility approaches.

The descriptive analysis found that the mean HSU derived from TTO and SG (0.95) were higher than those derived from the AQoL-7D (0.80) and VFQ-UI (0.83), which is consistent with previous studies that direct approaches tend to result in higher health utilities (reflecting better reported health) than indirect approaches for a wide range of diseases, and the difference can be substantial [33, 43]. The reason may be explained by methodological differences between the two approaches. Direct approaches need interviewers exchange some life years or take a risk for perfect health states, and capture the values that patients assign to their own health state. In contrast, indirect approaches are derived from algorithms that attribute a utility score to one’s health state based on the values of the general public [44]. Furthermore, it’s worth noting that both two direct approaches exhibited significant ceiling effect (TTO 7.5% & SG 20.5%), which means patients in the study were risk averse and unwilling to gamble and also unwilling to trade many life years. Patients’ preferences for health outcomes are affected by framing effects, contexts, anchoring points, duration of conditions, time preferences, attitudes towards risk and how life and health are valued by the respondent [45, 46]. Compared with other disease, patients with myopia may gradually learn to adapt to their situation and subjectively experience a relatively high HRQoL.

Two indirect approaches are moderate correlated in the analysis based on the Spearman’s ICC (> 0.5) and Bland–Altman plot. Although both them were demonstrated to be valid and sensitive in myopia patients, there were some important differences between two approaches. In terms of the concurrent validity, the VFQ-UI had stronger correlations than AQoL-7D with disease-specific instrument NEI VFQ-25, and “known-group” validation and sensitivity analysis further support the VFQ-UI had better discriminative ability than AQoL-7D. This is arguably due to differ in the dimensions described, the number of level, the severity range covered, and the concepts measured by the descriptive systems. The AQoL-7D, as a generic PBM, perceived general health rather than disease-specific quality of life, although it has 1 of its 7 dimensions and 10 of 26 items dedicated to ‘VisQoL’, which used to increase sensitivity for health states involving the loss of visual acuity and vision related handicap [19]. Nevertheless, vision function is only a subset of overall health state, any impact of visual impairment on generic HRQoL will be lost in the ‘noise’ of other nonvision-related impacts on HRQoL in the overall score. In contrast, the VFQ-UI is a very comprehensive measure of vision-related functioning and has the potential advantage of using a more sensitive descriptive system to classify people into health states [20].

The magnitude of ICC values ranging from -0.008 to 0.557, and the ICC between TTO and SG (0.111) is lower than that of AQoL-7D and VFQ-UI (0.557), which indicated there was a substantial lack of agreement between four health utility approaches in myopia. That may partly be due to the difference between the health state classification systems and the health state valuation methods [47]. In the indirect approaches, the HSU score was calculated based on a pre-defined value set derived from the general population. On the other hand, the direct approaches task elicited HSU score directly from individual patient’s perspective [48]. The overall poor agreements indicate that the choice of the approaches will have non-negligible effect on the HSU scores been elicited, and these four health utility approaches cannot be used interchangeably. In addition, the ICC can theoretically vary between 0 and 1.0, where an ICC of 0 indicates no reliability, whereas an ICC of 1.0 indicates perfect reliability [49]. Nevertheless, previous study has been confirmed that large negative values (less than -1) and large positive values (greater than + 1) are possible [50], which provide some explanations for the negative value of ICC (SG & AQoL-7D were -0.008, SG & VFQ-UI were -0.003) in our study.

The choice of health utility measures for the calculation of QALYs depends on many aspects, including but not limited to the psychometric properties of the measures, brevity and ease of use, the availability of country-specific tariff and the comparability across different diseases [51]. From an economics perspective, the SG and TTO are the most accepted approaches because they are based implicitly on utility theory and involve an inherent gamble or trade-off [52]. However, these approaches are relatively time-consuming and some patients have difficulties understanding the concept of probabilities. Meanwhile, the performance of SG and TTO were not as good as the AQoL-7D and VFQ-UI in the study. By comparison, the indirect approaches, especially PBMs are used more frequently, owing to their simple, require little explanation, less likely to suffer from ceiling effects, stratify data into a number of different dimensions, and stronger correlation with patient’s health states [33]. Considering the psychometric performance of four health utility approaches in myopic patients, the VFQ-UI, as a disease-specific PBM, has been validated in patients with glaucoma [53], diabetic macular edema [54], and other vision disorder conditions. It may be more suitable for application in clinical practice, given that it has only 6 items, easier and faster to complete, and comprehensively represents the patient’s perspective on the impact of ocular conditions on functioning and wellbeing [20]. Nevertheless, there are some disadvantages in the VFQ-UI, such as not be able to capture the impact of all side effects and comorbidities, and the values they generate are not directly comparable across different conditions [55]. Given the generic nature of the AQoL-7D, it is very important to use alongside the VFQ-UI to measure the generic dimensions of HRQoL for economic evaluations and health policy decisions.

There were some limitations to this study. Firstly, the study population of myopic patients was recruited at one tertiary hospital in eastern China. It may be interpreted as selective and not representative of all myopic patients in mainland China. Secondly, given there has not Chinese-specific tariff of AQoL-7D and VFQ-UI, we have used the only available official tariffs for calculating the health state utilities. Therefore, the results may differ from the health preferences of Chinese population. Thirdly, the cross-sectional design limits the ability to examine the responsiveness of the approaches. Further evidence on responsiveness for all four approaches in myopia patients should be explored. Forth, patients in this study were administered the four approaches simultaneously, and fatigue or boredom may have affected the quality of responses. The robustness of the results can be verified by reducing the number, or switching the order of the approaches in the future.

## Conclusions

Among the four health utility approaches investigated in this study, the VFQ-UI showed better psychometric properties than other three approaches for providing health state utility in myopia patients. Given the widespread use and its generic nature of the AQoL-7D, it could be used alongside with VFQ-UI to provide complementary health state utility from a generic and disease-specific perspective and a comprehensive measurement of HRQoL for economic evaluation and in subsequent decision making. More evidence on the responsiveness of four health utility approaches in myopia patients is required.

## Supplementary Information

**Additional file 1: Supplementary Table 1.** Participants’ HRQoL scores by each characteristic.

## Data Availability

The datasets used and/or analysed during the current study available from the corresponding author on reasonable request.

## Abbreviations

HRQoL: Health-related quality of life
CUA: Cost-utility analysis
QALYs: Quality-adjusted life years
HSU: Health states utility
SG: Standard gamble
TTO: Time trade-off
PBMs: Preference-based measures
HUI: Health Utility Index
AQoL: Assessment of Quality of Life
15D: 15 Dimensions
VFQ-UI: Visual Function Questionnaire–Utility Index
SE: Spherical equivalent
NEI VFQ-25: National Eye Institute Visual Functioning Questionnaire-25
SD: Standard deviation
ES: Effect size
RE: Relative efficiency
AUC: Area under the receiver operating characteristic curve
ICC: Intra-class correlation coefficient
LOA: Limits of agreement
