# Supplementary material for: Psychometric properties and comparison of four health utility approaches among myopia patients in China
Source: Health Qual Life Outcomes. 2023 Jul 4;21:66. doi: 10.1186/s12955-023-02150-w (PMC10320907; doi:10.1186/s12955-023-02150-w)
Supplement: Supplementary file 1 — Additional file 1: Supplementary Table 1. Participants’ HRQoL scores by each characteristic. [file 12955_2023_2150_MOESM1_ESM.docx]

**Supplementary Table1 Participants’ HRQoL scores by each characteristic**

| **Characteristics** | **TTO** | | **SG** | | **AQoL-7D** | | **VFQ-UI** | |
| --- | --- | --- | --- | --- | --- | --- | --- | --- |
|  | **Mean (SD)** | ***P*-value** | **Mean (SD)** | ***P*-value** | **Mean (SD)** | ***P*-value** | **Mean (SD)** | ***P*-value** |
| **Age (Years)** |  |  |  |  |  |  |  |  |
| **≤25** | 0.95 (0.05) | 0.732 | 0.93 (0.11) | 0.000 | 0.80 (0.11) | 0.633 | 0.83 (0.10) | 0.001 |
| **＞25** | 0.95 (0.07) |  | 0.97 (0.07) |  | 0.80 (0.11) |  | 0.84 (0.10) |  |
| **Gender** |  |  |  |  |  |  |  |  |
| Male | 0.95 (0.06) | 0.271 | 0.94 (0.10) | 0.035 | 0.80 (0.11) | 0.627 | 0.83 (0.10) | 0.407 |
| Female | 0.95 (0.06) |  | 0.96 (0.10) |  | 0.80 (0.12) |  | 0.84 (0.10) |  |
| **Occupation** |  |  |  |  |  |  |  |  |
| Students | 0.95 (0.06) | 0.874 | 0.93(0.11) | 0.000 | 0.80 (0.11) | 0.371 | 0.82 (0.10) | 0.003 |
| Others | 0.95 (0.06) |  | 0.96 (0.09) |  | 0.80 (0.11) |  | 0.84 (0.10) |  |
| **Educational level** |  |  |  |  |  |  |  |  |
| Primary or secondary school | 0.95 (0.11) | 0.476 | 0.93 (0.09) | 0.000 | 0.77 (0.15) | 0.043 | 0.78 (0.12) | 0.004 |
| High school or technical secondary school | 0.95 (0.05) |  | 0.92 (0.12) |  | 0.78 (0.12) |  | 0.82 (0.10) |  |
| Junior college | 0.95 (0.05) |  | 0.95 (0.08) |  | 0.80 (0.12) |  | 0.84 (0.10) |  |
| University degree and above | 0.95 (0.06) |  | 0.96 (0.09) |  | 0.82 (0.10) |  | 0.85 (0.10) |  |
| **Marital status** |  |  |  |  |  |  |  |  |
| Married | 0.95 (0.07) | 0.192 | 0.96 (0.07) | 0.013 | 0.80 (0.12) | 0.906 | 0.84 (0.10) | 0.003 |
| Single | 0.95 (0.05) |  | 0.94 (0.11) |  | 0.80 (0.11) |  | 0.83 (0.10) |  |
| **Residence** |  |  |  |  |  |  |  |  |
| Rural | 0.95 (0.07) | 0.530 | 0.95 (0.08) | 0.200 | 0.80 (0.12) | 0.767 | 0.83 (0.10) | 0.536 |
| Urban | 0.95 (0.06) |  | 0.95 (0.10) |  | 0.80 (0.11) |  | 0.83 (0.10) |  |
| **Severity of myopia** |  |  |  |  |  |  |  |  |
| Low | 0.95 (0.05) | 0.761 | 0.94 (0.12) | 0.278 | 0.81 (0.11) | 0.200 | 0.85 (0.09) | 0.001 |
| Moderate to High | 0.95 (0.06) |  | 0.95 (0.09) |  | 0.80 (0.11) |  | 0.83 (0.10) |  |
| **Duration of myopia (Years)** |  |  |  |  |  |  |  |  |
| ≤10 | 0.95 (0.05) | 0.053 | 0.95 (0.10) | 0.423 | 0.81 (0.10) | 0.269 | 0.83 (0.10) | 0.000 |
| ＞10 | 0.94 (0.07) |  | 0.95 (0.10) |  | 0.79 (0.12) |  | 0.79 (0.12) |  |

SD, standard deviation.
